# Sexual violence in older adults: a Belgian prevalence study

**DOI:** 10.1186/s12877-021-02485-3

**Published:** 2021-10-26

**Authors:** Anne Nobels, Adina Cismaru-Inescu, Laurent Nisen, Bastien Hahaut, Marie Beaulieu, Gilbert Lemmens, Stéphane Adam, Evelyn Schapansky, Christophe Vandeviver, Ines Keygnaert

**Affiliations:** 1 International Centre for Reproductive Health, Department of Public Health and Primary Care, Ghent University, Ghent, Belgium; 2 Department of Psychiatry, Ghent University Hospital, Ghent, Belgium; 3 CARE-ESPRIst, Études et évaluations, University of Liège, Liège, Belgium; 4 Psychology of Aging Unit, University of Liège, Liège, Belgium; 5 School of Social Work and Research Centre on Aging, University of Sherbrooke, Quebec, Canada; 6 Department of Head and Skin – Psychiatry and Medical Psychology, Ghent University, Ghent, Belgium; 7 Department of Criminology, Criminal Law and Social Law, Ghent University, Ghent, Belgium; 8 Research Foundation—Flanders (FWO), Brussels, Belgium

**Keywords:** Sexual abuse, Sexual assault, Elder abuse and neglect, Ageing

## Abstract

**Background:**

Sexual violence (SV) is an important public health problem which may cause long-lasting health problems. SV in older adults remains neglected in research, policies and practices. Valid SV prevalence estimates and associated risk factors in older adults are currently unavailable. In this study we measured lifetime and past 12-months sexual victimisation in older adults living in Belgium, its correlates, assailant characteristics and the way that victims framed their SV experiences.

**Methods:**

SV was measured using behaviourally specific questions based on a broad definition of SV. Participants were selected via a cluster random probability sampling with a random route finding approach. Information on sexual victimisation, correlates, assailant characteristics and framing was collected via structured face-to-face interviews with adults aged 70 years and older living in Belgium (community-dwelling, assisted living and nursing homes).

**Results:**

Among the 513 participants, the lifetime SV prevalence was 44% (55% F, 29% M). Past 12-months prevalence was 8% (9% F, 8% M). Female sex and a higher number of sexual partners were associated with lifetime SV (*p* < .05), non-heterosexual sexual orientation with past 12-months SV (*p* < .05). Correlates identified to be linked to elder abuse and neglect in previous studies were not linked with SV in our sample. ‘Someone unknown’ was identified as most common assailant.

**Conclusions:**

Sexual victimisation appears to be common in older adults in Belgium. Both correlates and assailant characteristics seem to differ from previous studies on elder abuse and neglect. Recognizing older adults as a risk group for sexual victimisation in research, policies and practices is of the utmost importance.

**Supplementary Information:**

The online version contains supplementary material available at 10.1186/s12877-021-02485-3.

## Background

Sexual violence (SV) [1] is increasingly considered a public health problem of major societal and judicial concern [2, 3]. Extensive research links sexual victimisation to long-lasting sexual, reproductive, physical, and mental health problems [2–4]. Exposure to childhood sexual abuse has been linked to depression, anxiety, and somatic complaints in older adults [5, 6].

Previous research suggests that SV in older adults rarely occurs [7]. A recent meta-analysis showed that 0.9% of community-dwelling older adults worldwide were sexually victimised in the past 12-months [8]. In Europe, numbers of past 12-months SV prevalence in older adults varied between 0 and 3.1% [9]. In a Belgian study, lifetime SV prevalence was estimated at 6.3% [10]. However, current studies show low SV prevalence numbers as they conflate it with other types of violence in the broader context of elder abuse and neglect [9], domestic violence or intimate partner violence [11]. Studies exclusively focussing on SV in older adults, describe criminal cases, and judicial response [12, 13]. Yet, research on SV in older adults from a public health perspective, providing valid SV prevalence numbers and correlates, is currently lacking. Several risk factors have been associated with elder abuse and neglect, such as poor (perceived) health status, care dependency, low social support, and financial strain [14–19]. However, specific risk factors for sexual violence in old age are currently unknown [9].

Assessing sexual victimisation in older adults may be challenging for myriad reasons. Older adults grew up in a time when talking about sexuality and SV was considered taboo. They may also have different perceptions of sexuality and SV compared to younger generations [20], because of limited sexual education when they were young, different legal definitions and ideas on sexual consent [21, 22]. Furthermore, older adults are considered asexual by society [23]. Internalizing this stereotypical image of ‘the asexual older adult’, they may not identify themselves as possible SV victims [24, 25], which could lead to a reluctance to disclose sexual victimisation, and to seek help [26, 27]. Moreover, health care workers feel that sexuality and SV are not legitimate topics to discuss with older adults and are worried to offend their patients when they do so [28, 29]. Also, they seem to have insufficient communication skills to adequately deal with SV in later life [28].

In spite of the call by the United Nations to significantly reduce all forms of violence [29], policies on SV in older adults are currently non-existent [9]. In order to develop preventive measures and to provide tailored care for older SV victims, a revision of current policies and health care practices is of the utmost importance [9, 30]. To make this possible, a better understanding of the prevalence and nature of SV in older adults is crucial. To our knowledge, this study is the first in its kind to assess lifetime and past 12-months sexual victimisation, correlates, assailant characteristics and the way that older victims framed their SV experiences. Based on the results, we identify avenues for future research, and formulate recommendations for policies and health care practices.

## Methods

### Measures

We adopted the WHO definition of SV, which includes different forms of sexual harassment without physical contact (hands-off SV), sexual abuse with physical contact but without penetration and (attempted) rape (hands-on SV) [1, 3]. This definition was expanded to include sexual neglect, as a result of recent insights in the field of SV in older adults [9, 31]. In 2017, a group of scientists, professionals and policy makers from Quebec, Canada, defined sexual neglect as ‘a failure to provide privacy, a failure to respect a person’s sexual orientation or gender identity, treating older adults as asexual beings and/ or preventing them from expressing their sexuality, etc.’ [31].

This study was a part of a national SV prevalence study in the Belgian population between 16 and 100 years old. We used the same questionnaire across all ages. The questionnaire development comprised a multi-step process of discussion and consultation between the multidisciplinary research team and an expert steering committee consisting of national and international researchers, policy makers and practitioners in the field of SV or elder abuse and neglect. An extensive description of the validation of the questionnaire in the Belgian population, including older adults, is available elsewhere [32, 33]. Moreover, we conducted a two-phase pilot study to test the acceptability and feasibility of the questionnaire in older adults. More details on this pilot study were published elsewhere [34]. Participants were asked, among others, questions on sociodemographic characteristics, sexual health & relations and sexual victimisation. In order to provide valid estimates of both female and male sexual victimisation, we used behaviourally specific questions (BSQ) to assess lifetime and past 12-months SV experiences [35]. The SV items were based on existing surveys [36–38], and adapted to the Belgian social and legal context [32, 33]. Due to the absence of a standardised measure for sexual neglect, it was assessed as “touching in care” (see Appendix 1). Participants who indicated to have experienced lifetime SV, were asked further questions on the assailant(s) and how they framed their sexual violence experience(s). For hands-off SV questions were asked for the behaviour that impacted the victim the most. For hands-on SV, these questions were asked for each of the SV behaviours. Moreover, for each hands-on SV behaviour, victims were asked to indicate the types of coercion strategies that were used. The questions on assailants, framing and coercion strategies are available in Appendices 2, 3 and 4.

### Sample selection

Between the 8th July 2019 and the 12th March 2020, 513 older adults across Belgium were interviewed. Based on our power analysis, the target sample size was 845 participants [34]. It was anticipated this sample size would provide a SV prevalence estimate with a 3 % margin of error. However, the COVID-19 pandemic and associated lockdown measures forced us to prematurely stop data collection. Cluster random probability sampling was used to obtain representative results for the Belgian older population. Eligible participants were identified using a random walk procedure [34, 39]. Participants had to be at least 70 years old, live in Belgium, speak Dutch, French or English, and have sufficient cognitive ability to complete the interview. Cognitive status was assessed based on the ability to maintain attention during the interview and the consistency of the participant’s answer, by means of a control question comparing the participant’s birth year and age. Both older adults living in the community and living in nursing homes or assisted living facilities were included. Face-to-face interviews were carried out by trained interviewers in private at the participant’s place of residence.

The study was conducted according to the Declaration of Helsinki and the WHO ethical and safety recommendations for SV research [40], and received ethical approval from the ethical committee of Ghent University/University Hospital (Belgian reference number: B670201837542). All participants gave their written informed consent before participating in the study. After participation they were given the contact details of several helplines. Participation rate was 34%. The full study protocol is available elsewhere [34].

### Analyses

Statistical analysis was performed using R version 3.6.3 and SPSS Statistics 26. The 17 SV variables were grouped into hands-off (eight items) and hands-on SV (nine items), the latter being further grouped into sexual abuse (four items) and attempted or completed rape (five items). For the purpose of the analysis the item measuring sexual neglect was grouped under sexual abuse. We created dichotomous variables out of all items in order to assess lifetime and past 12-months victimisation. A detailed overview of the SV outcome measures can be found in Appendix 1.

A number of demographic and socio-economic variables and variables related to the participants’ sexual health and relations were included in the multivariate logistic regression analysis. All variables were added simultaneously. Adjusted odds ratios describe the correlation with sexual victimisation while adjusting for the other variables in the model. The multi-collinearity assumption of multivariate regression analyses was tested with the Variance Inflation Factor (VIF) and indicated no violation. Social support (measured by number of confidants) could be added as a continuous variable into the model without violating the linearity assumption. The number of lifetime sexual partners and age of sexual initiation were recoded into dichotomous variables based on the median.

## Results

### Study population characteristics

The study sample consisted of a valid representation of the Belgian population aged 70 years and older [34]. The mean age was 79 years (SD: 6.4 yrs., range 70-99 yrs), 58.3% was female, 89.8% was community-dwelling, 90.4% was born in Belgium, 31.2% completed higher education, 50.3% was in a relationship and 7.4% labelled themselves as non-heterosexual. This group contains participants who labelled themselves as homosexual, bisexual, pansexual, asexual or other. In this last group, several participants labelled themselves as “normal”. Since it was not clear whether they had difficulties understanding the different terms defining sexual orientation or whether they indeed labelled their sexual orientation as “other”, we decided to classify these participants as non-heterosexual. More information on the sample composition in comparison to the Belgian population of 70 years and older can be found in Appendix 5.

### Prevalence of sexual victimisation

The lifetime prevalence of SV was 44.2% (95% CI: 39.9–48.7), 55.2% (95% CI: 49.4–60.9) of females and 29.0% (95% CI: 23.0–35.5) of males. Almost half of women and one in four men experienced hands-off SV, one in three women and one in six men reported hands-on SV. One in twelve females and one in 30 males disclosed an (attempted) rape. In the past 12-months, 8.4% (95% CI: 6.1–11.1) experienced at least one form of SV, 9% of females and 7.5% of males. Hands-off SV was reported by 7.7% of females and 6.1% of males, hands-on SV by 2.7% of females and 2.3% of males. The most commonly reported sexually transgressive behaviours were unwanted sexual staring, sexual innuendo and kissing; both during lifetime and in the past 12-months.

A more detailed description of the prevalence of all different forms of SV can be found in Table 1.

**Table 1:** Detailed lifetime and past 12-months prevalence sexual victimisation, by sex

	Men (*n* = 214)		Women (*n* = 299)		Total (n = 513)	
Item	Lifetime % (95% CI)	Past 12-months % (95% CI)	Lifetime % (95% CI)	Past 12-months % (95% CI)	Lifetime % (95% CI)	Past 12-months % (95% CI)
**Any SV**	**29.0 (23.0–35.5)**	**7.5 (4.3–11.9)**	**55.2 (49.4–60.9)**	**9.0 (6.0–12.9)**	**44.2 (39.9–48.7)**	**8.4 (6.1–11.1)**
**Any Hands-Off SV**	**22.4 (17.0–28.6)**	**6.1 (3.3–10.2)**	**45.2 (36.4–51.0)**	**7.7 (4.9–11.3)**	**35.7 (31.5–40.0)**	**7.0 (5.0–9.6)**
Sexual staring	11.2 (7.3–16.2)	2.3 (0.8–5.4)	23.7 (19.0–29.0)	2.7 (1.2–5.2)	18.5 (15.2–22.2)	2.5 (1.4–4.3)
Sexual innuendo	7.0 (4.0–11.3)	3.3 (1.3–6.6)	22.4 (17.8–27.6)	3.0 (1.4–5.6)	16.0 (12.9–19.5)	3.1 (1.8–5.0)
Showing sexual images	5.1 (2.6–9.1)	2.3 (0.8–5.4)	6.4 (3.9–9.7)	0.7 (0.1–2.4)	5.9 (4.0–8.3)	1.4 (0.6–2.8)
Sexual calls or texts	4.2 (1.9–7.8)	1.4 (0.3–4.0)	8.0 (5.2–11.8)	1.3 (0.4–3.4)	6.5 (4.5–9.0)	1.4 (0.6–2.8)
Voyeurism	0.5 (0.0–2.6)	0.0 (0.0–1.7)	0.3 (0.0–1.9)	0.0 (0.0–1.2)	0.4 (0.0–1.4)	0.0 (0.0–0.7)
Distributing sexual images	0.0 (0.0–1.7)	0.0 (0.0–1.7)	0.0 (0.0–1.2)	0.0 (0.0–1.2)	0.0 (0.0–0.7)	0.0 (0.0–0.7)
Exhibitionism	5.6 (2.9–9.6)	1.4 (0.3–4.0)	20.7 (16.3–25.8)	1.7 (0.5–3.9)	14.5 (11.5–17.8)	1.6 (0.7–3.0)
Forcing to show intimate body parts	1.9 (0.5–4.8)	0.0 (0.0–1.7)	3.0 (1.4–5.6)	0.7 (0.1–2.4)	2.5 (1.4–4.3)	0.4 (0.0–1.4)
**Any Hands-On SV**	**15.9 (11.3–21.5)**	**2.3 (0.8–5.4)**	**35.1 (29.7–40.8)**	**2.7 (1.2–5.2)**	**27.1 (23.3–31.2)**	**2.5 (1.4–4.3)**
**Any Sexual Abuse**	**13.6 (9.3–18.9)**	**2.3 (0.8–5.4)**	**33.8 (28.4–39.4)**	**2.3 (0.9–4.8)**	**25.3 (21.6–29.3)**	**2.3 (1.2–4.1)**
Kissing	8.9 (5.4–13.5)	1.9 (0.5–4.7)	21.1 (16.6–26.6)	1.7 (0.5–3.9)	16.0 (12.9–19.4)	1.8 (0.8–3.3)
Touching in care	0.9 (0.1–3.3)	0.5 (0.0–2.6)	5.4 (3.1–8.5)	0.7 (0.1–2.4)	3.5 (2.1–5.5)	0.6 (0.1–1.7)
Fondling/rubbing	6.1 (3.3–10.2)	1.9 (0.5–4.7)	16.4 (12.4–21.1)	1.7 (0.5–3.9)	12.1 (9.4–15.2)	1.8 (0.8–3.3)
Forced undressing	1.9 (0.5–4.7)	1.9 (0.5–4.7)	3.0 (1.4–5.6)	1.7 (0.5–3.9)	2.5 (1.4–4.3)	1.8 (0.8–3.3)
**Any Rape**	**3.3 (1.3–6.6)**	**0.0 (0.0–1.7)**	**8.4 (5.5–12.1)**	**1.0 (0.2–2.9)**	**6.2 (4.3–8.7)**	**0.6 (0.1–1.7)**
Oral penetration	0.5 (0.0–2.6)	0.0 (0.0–1.7)	1.7 (0.5–3.9)	0.0 (0.0–1.2)	1.2 (0.4–2.5)	0.0 (0.0–0.7)
Attempt of oral penetration	1.4 (0.3–4.0)	0.0 (0.0–1.7)	3.3 (1.6–6.1)	0.3 (0.0–1.8)	2.5 (1.4–4.3)	0.2 (0.0–1.1)
Vaginal or anal penetration	0.9 (0.1–3.3)	0.0 (0.0–1.7)	4.3 (2.3–7.3)	0.3 (0.0–1.8)	2.9 (1.6–4.8)	0.2 (0.0–1.1)
Attempt of vag. or anal penetr.	0.9 (0.1–3.3)	0.0 (0.0–1.7)	2.0 (0.7–4.3)	0.3 (0.0–1.8)	1.6 (0.7–3.1)	0.2 (0.0–1.1)
Forcing to penetrate	0.0 (0.0–1.7)	0.0 (0.0–1.7)	0.3 (0.0–1.8)	0.0 (0.0–1.2)	0.2 (0.0–1.1)	0.0 (0.0–0.7)

Abbreviations: *SV* Sexual Violence, *CI* Confidence Interval

### Coercion strategies

**Fig. 1:**
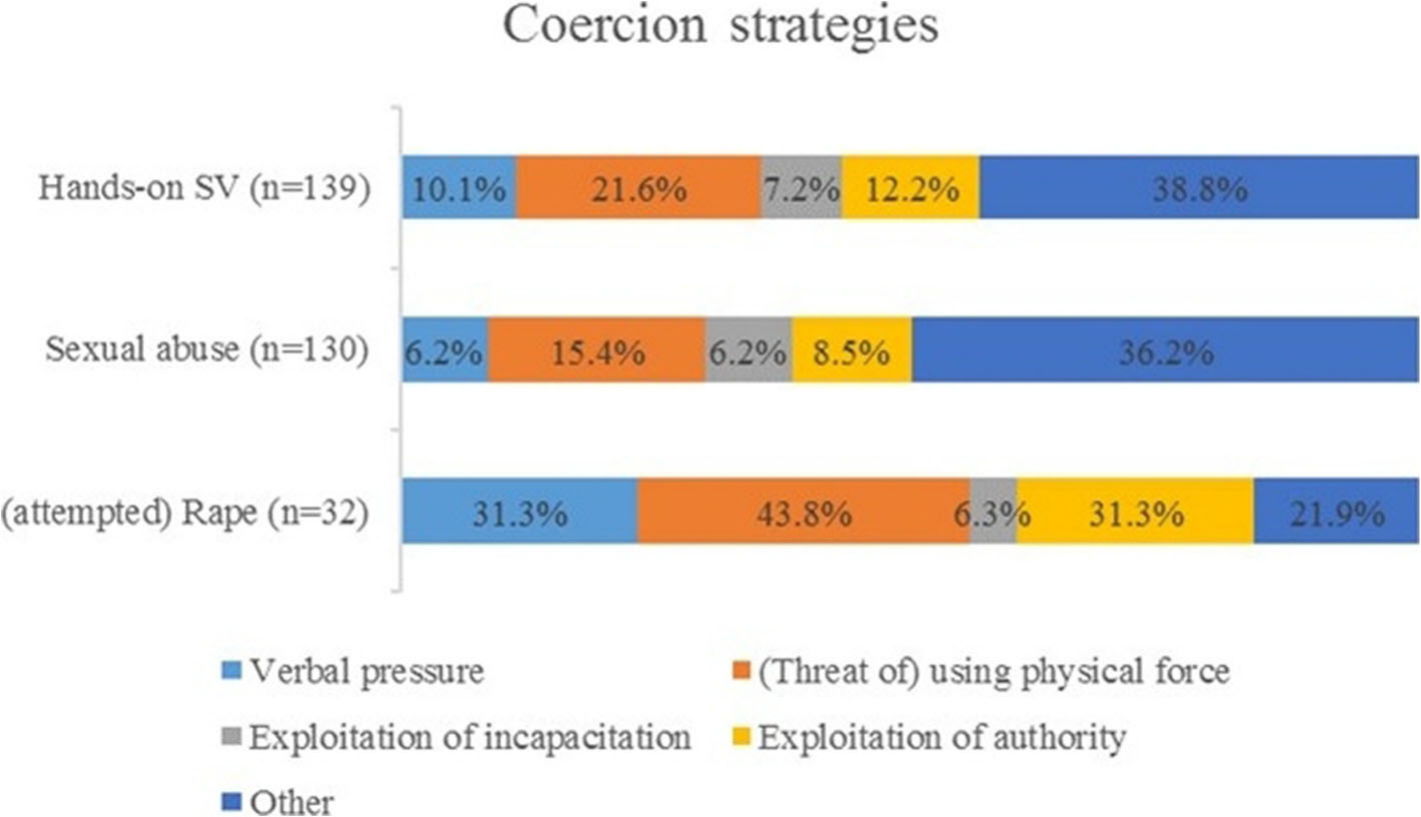
**Type of coercion used for hands-on lifetime sexual violence, sexual abuse, and (attempted) rape**. Note. Respondents could provide multiple answers, unless “Other” = None of the above was selected. Abbreviations: SV = sexual violence

Figure 1 shows the types of coercion used by the assailants for the different types of hands-on lifetime SV. Over one third of the victims indicated that none of the provided response options applied to their situation. For (attempted) rape specifically, (threat of) using physical force was the most commonly identified coercion strategy.

### Characteristics of SV victims

Table 2 summarizes the results of the logistic regression analysis. Women were more likely to be sexually victimised in their lifetime, but for the past 12-months we found no difference between women and men regarding sexual victimisation. Participants with two or more lifetime sexual partners experienced more SV in their life compared to participants with fewer than two sexual partners. This difference was not significant in the past 12-months. Regarding sexual orientation, we found that older adults who identified themselves as non-heterosexual experienced significantly more SV in the past 12-months. However, for lifetime SV this difference was not significant.

**Table 2:** Sexual victimisation correlates

		Lifetime SV	Past 12-months SV
Predictors		aOR (95% CI)	aOR (95% CI)
Sex	Female	3.60 (2.35–5.52) *	1.57 (0.74–3.34)
	Male	Ref	Ref
Perceived age	Younger	1.43 (0.87–2.36)	0.85 (0.36–2.00)
	Same	Ref	Ref
	Older	0.85 (0.29–2.46)	/
Sexual orientation	Heterosexual	Ref	Ref
	Non-heterosexual	0.80 (0.38–1.70)	3.23 (1.17–8.94) *
Living situation	Community-dwelling	Ref	Ref
	Assisted living	2.01 (0.78–5.20)	0.97 (0.20–4.62)
	Nursing home	0.70 (0.26–1.91)	0.94 (0.19–4.85)
Relationship status	No partner	Ref	Ref
	Not living with partner	0.96 (0.62–1.49)	0.51 (0.23–1.14)
	Living with partner	0.67 (0.30–1.53)	0.21 (0.03–1.69)
Education level	Primary or none	0.75 (0.44–1.29)	0.60 (0.24–1.52)
	Secondary	0.87 (0.55–1.36)	0.54 (0.25–1.19)
	Higher	Ref	Ref
Financial status	Easy	Ref	Ref
	Difficult	1.02 (0.66–1.60)	0.67 (0.29–1.55)
Care dependency	No	Ref	Ref
	Yes	1.04 (0.68–1.59)	0.91 (0.43–1.94)
Social support		1.01 (0.99–1.04)	1.00 (0.96–1.04)
Perceived health status	No disability/chronical illness	Ref	Ref
	Disability/chronical illness	0.96 (0.64–1.45)	0.84 (0.41–1.74)
Sexual initiation^a^	Early (< 21 years)	1.25 (0.84–1.86)	1.16 (0.56–2.39)
	Late (≥21 years)	Ref	Ref
N of lifetime sexual partners^a^	< 2	Ref	Ref
	≥ 2	1.54 (1.01–2.34)*	1.93 (0.92–4.04)

Abbreviations: *SV* Sexual violence, *aOR* adjusted odds ratio

*p < .05

^a^Sexual initiation and number of lifetime sexual partners were dichotomized based on the median

### Assailant characteristics

**Fig. 2:**
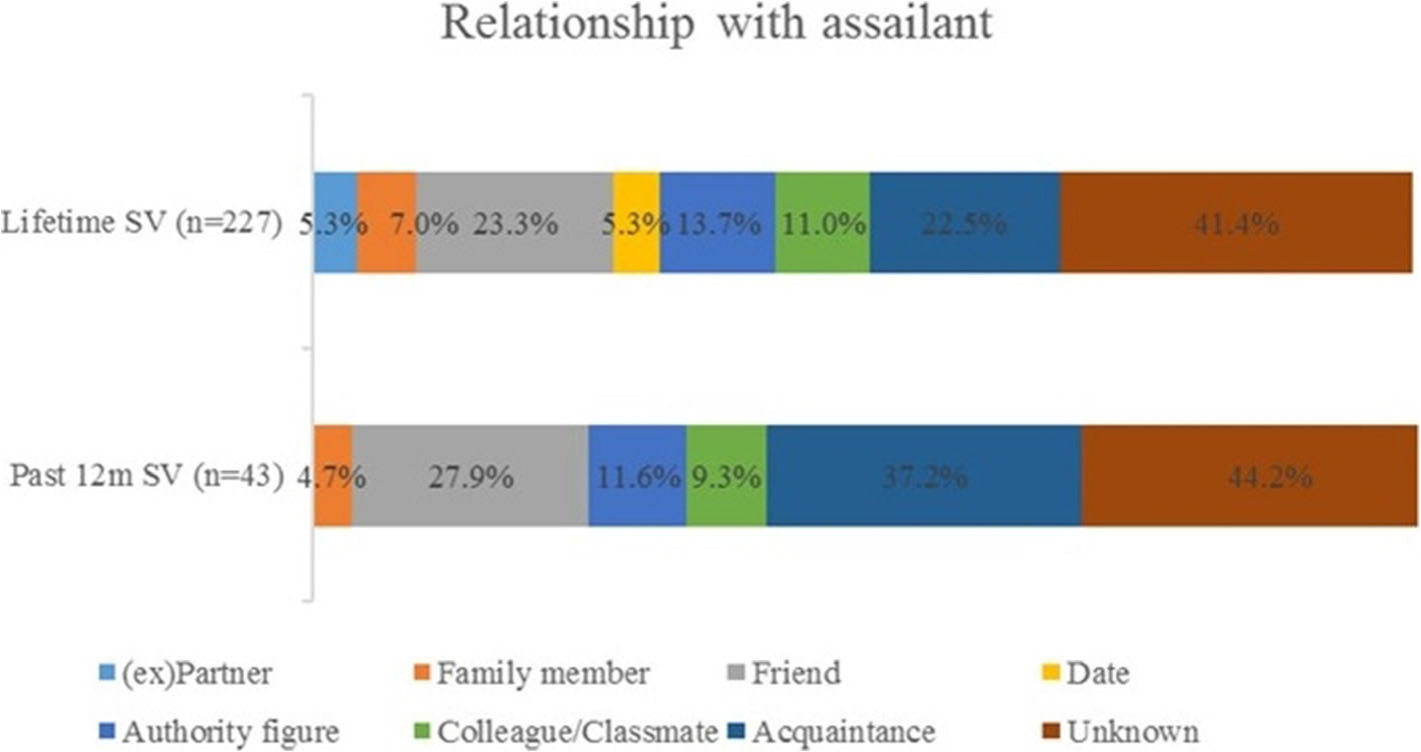
**Relationship between victim and assailant of sexual violence, in %.** Note. Participants could provide multiple answers. Abbreviations: SV = sexual violence, past 12 m = past 12-months

For lifetime SV, 83.6% of assailants were male, 15.0% were female, and in 1.4% of the cases the sex of the assailant was unknown. In the past 12-months, 73.3% of assailants were male, 24.4% were female and in 0.2% of the cases the sex of the assailant was unknown. Mean age of the assailant committing SV in the past 12-months, as estimated by the victim, was 48.9 years (SD 18.9 yrs). For both lifetime and past 12-months SV ‘someone unknown’ was most often identified as the assailant, respectively in 41.4 and 44.2% of the cases. More details on the relationship between victim and assailant can be found in Fig. 2.

### Framing of sexual violence by victims

Table 3 summarizes how victims of lifetime SV framed their experience(s). In 47.6% of the cases, SV was framed as ‘just something that happened’, in 34.4% as ‘wrong, but not a crime’ and in 23.3% as a crime. Concerning rape, we found that in 28.1% of cases victims framed it as ‘just something that happened’, in 28.1% as ‘wrong, but not a crime’, and in 43.8% as a crime.

**Table 3:** Framing of lifetime sexual violence by victims

	A Crime	Wrong but not a crime	Just something that happened
	n (%)	n (%)	n (%)
Any SV (*n* = 227)	53 (23.3)	78 (34.4)	108 (47.6)
Any hands-off (*n* = 183)	34 (18.6)	53 (23.0)	72 (26.8)
Any hands-on (*n* = 139)	34 (24.5)	42 (38.1)	49 (51.8)
Sexual Abuse (*n* = 130)	27 (20.8)	47 (36.2)	68 (52.3)
(attempted) Rape (*n* = 32)	14 (43.8)	9 (28.1)	9 (28.1)

Note. For hands-off SV only the incident with the most impact on the victim was included in the analysis. For hands-on SV all incidents were included and grouped into sexual abuse, (attempted) rape and hands-on SV. If victims indicated a different framing for different incidents, they are included as separate answers and so the total % is > 100%

Abbreviations: SV = sexual violence

## Discussion

In this paper we present a Belgian prevalence study on sexual victimisation in older adults. We conducted 513 interviews with people aged 70 years and older across Belgium.

Our results show that lifetime exposure to SV is highly prevalent among older adults in Belgium. Over 44% of participants were sexually victimized during their lifetime. Despite the assumption that older adults are at low risk for sexual victimisation [7], in our study, one in 12 older adults experienced at least one form of SV in the past 12-months. Our numbers appear higher compared to previous European studies in community-dwelling older adults in which the estimated lifetime SV prevalence was 6.3% and the past 12-months prevalence rates varied between 0 and 3.1% [9, 10]. This difference could be explained by several methodological choices. First, we studied SV in older adults from a different perspective compared to previous studies that researched SV based on criminal cases [12, 13] or as a form of elder abuse and neglect, domestic violence or intimate partner violence [9, 11]. Hence, they restricted the relation between victim and assailant to a confidant, a household member or an intimate partner respectively while our research shows that assailants are also unknown. Moreover, previous research only included forms of hands-on SV (sexual abuse with physical contact and (attempted) rape). Applying the broad WHO definition of SV, we included both hands-off and hands-on SV regardless of the relation between victim and assailant, leading to increased lifetime and past 12-months SV prevalence numbers. Second, the use of the BSQ made it easier for participants to remember and engage with the situations presented. Furthermore, BSQ leave less room for interpretation, stigma or labelling which makes it possible for people who do not identify as a victim to indicate their SV experiences, leading to more valid estimates [35].

However, compared to an online study in the Belgian population aged 16 to 69 years using the same questionnaire, we found lower lifetime and past 12-months prevalence rates [32]. This decreased SV reporting with increasing age may adequately represent lower sexual victimisation rates in older adults or may be explained by several factors, such as reduced recall in general [41], reduced recall of negative events [42, 43], or higher mortality among people with a SV history [44, 45]. Moreover, older adults might have a different perception of SV than younger generations. In our study, in 47.6% of SV cases and in 28.1% of rape cases, victims perceived it as ‘just something that happened’. Previous studies found that generational specificities surrounding sexuality and SV such as legal definitions and perceptions of SV, influenced disclosure rates [13, 46]. Furthermore, society’s attitudes regarding sexuality have become more permissive, and the definition of sexual consent has been narrowed [20]. For example, until the end of the twentieth century being married implied consent to sexual intercourse, whereas today spousal rape is considered a criminal offence [21]. In our study, in only 5.3% of lifetime SV cases, the (ex) partner was identified as assailant, which is much less compared to studies in younger populations in which over 25% of women indicated being sexually victimised by their (ex) partner [47]. Finally, because of the image of ‘the asexual older adult ‘[23], older adults might not identify themselves as a victim of SV [24, 25]. In previous studies on elder abuse and neglect, older adults did not acknowledge SV as a possible form of abuse [48]. To a certain extent, we have pre-emptively addressed this by adopting BSQ to measure sexual victimisation, as BSQ allow victims who do not identify themselves as such to indicate their experiences [35]. Nevertheless, such beliefs may have inadvertently influenced SV disclosure in our study.

In addition to measuring SV prevalence, our study aimed to provide an analysis of SV correlates in older adults. Correlates linked to elder abuse and neglect in previous studies, such as poor (perceived) health status, care dependency, low social support, and financial strain [14–19], were not associated with sexual victimisation in our sample. We assume this can be explained by the use of a broad definition of SV including both hands-off and hands-on SV, which is different from the definitions used in studies on elder abuse and neglect. Moreover, there is a possible underpower of our sample which could explain the low number of correlates identified. Being female and having a greater number of lifetime sexual partners were associated with lifetime sexual victimisation, which is in line with previous research on SV in younger populations [37, 49]. For past 12-months SV, we could not identify a difference between men and women. Previous research showed inconclusive results. Although some studies described older women as being more prone to SV [50, 51], others showed that women and men were equally at risk [52, 53]. In our sample, being non-heterosexual was correlated to past 12-months SV. Previous research has linked LGBT+ status, often intertwined with other factors such as disability and poverty, to intimate partner violence among older adults [54], but for SV this has not been reported before. However, our results have to be interpreted with caution as a possible difficulty of several participants to understand the different terms defining sexual orientation, could lead to an overestimation of non-heterosexual people in our sample. Furthermore, our results confirm previous findings that assailants of SV in older adults tend to be younger than the victim [50].

Regarding coercion strategies, we found that the (threat of) using physical force was the most common coercion used for any type of rape. For any type of sexual abuse, over one third of the participants indicated that none of the mentioned types of coercion were used. This suggests our study did not capture all types of coercion strategies used in hands-on SV [32, 33]. A recent study by Canan et al. [55] in victims from 23 to 68 years old identified more coercion strategies, such as ‘making me feel as though refusing was useless’ and ‘just doing the behaviour without giving me a chance to say “no” (e.g., surprising me with the behaviour).’ More research is needed to check whether these coercion strategies can be extrapolated to SV in old age. Previous studies showed inconclusive results regarding coercion strategies. Although some studies reported physical force was more often used on older SV victims compared to younger victims, most studies did not report significant differences between younger and older victims regarding use of physical force as a coercion strategy [12]. Because our findings are similar to the coercion strategies identified by younger victims in Belgium [32], we assume that the coercion strategies used on older adults are similar to the ones used on younger victims and not as violent as believed [12].

An important limitation of our study was that the target sample size of 845 interviews could not be reached due to the COVID-19 pandemic and associated lockdown measures. However, the current sample size of 513 interviews allowed us to report on prevalence rates within 4 % of the estimated value. Furthermore, due to the absence of a standardised measure for sexual neglect, we narrowed it down to “touching in care” which is an incomplete representation of the definition [31] and supposed reality. Nevertheless, this study is, to our knowledge, the first of its kind to measure the prevalence, correlates, assailant characteristics and framing of SV in older adults. It can be regarded as an important step towards a better understanding of the magnitude, nature and impact of SV in older adults. Responding to the call of Bows [12] to consider SV as a particular form of violence in old age and study it independently from other forms of elder abuse and neglect and domestic violence, this study brings a new perspective on SV in older adults. For future studies, we encourage the development of measurement tools for sexual neglect in order to incorporate this form of SV as well.

Based on our findings we reinforce previous recommendations for policy makers to recognise older adults as a risk group for sexual victimisation [12]. Furthermore, our study showed that assessing SV in older adults is possible without offending them [34]. Professionals urgently need capacity building to better detect signs, prevent, mitigate and respond to SV in old age. Finally, sensitisation of society in general is essential, emphasizing the prevention of SV against older adults.

## Conclusions

Sexual victimisation appears to be common in older adults in Belgium. Over 44% experienced SV in their lifetime and one in 12 in the past 12-months. Being female and having had a greater number of lifetime sexual partners were linked to lifetime SV, a non-heterosexual sexual orientation to past 12-months victimisation. Correlates generally linked to elder abuse and neglect did not seem to be linked with SV. Our findings highlight the importance of recognising older adults as a risk group for sexual victimisation and to study SV independently from other forms of violence in old age. In order to detect signs, prevent, mitigate and respond to SV in older adults, sensitisation of society and capacity building of professionals is needed.

## Supplementary Information

**Additional file 1: Appendix 1.** Detailed outcome measurements sexual victimisation. **Appendix 2.** Question on assailants of sexual violence. **Appendix 3.** Question on framing of sexual violence. **Appendix 4.** Question regarding coercion strategies. **Appendix 5.** Sociodemographic characteristics of the study population (*n* = 513) compared to the Belgian population of 70 years and older.

## Data Availability

The datasets used and analysed during the current study are available from the corresponding author on reasonable request.

## Abbreviations

aOR: Adjusted odds ratio
BSQ: Behaviourally specific questions
CI: Confidence interval
LGBT+: Lesbian gay bisexual transgender plus
SD: Standard deviation
SV: Sexual violence
UN: United Nations
VIF: Variance Inflation Factor
WHO: World Health Organization
