# Correction to: Sexual violence in older adults: a Belgian prevalence study

**DOI:** 10.1186/s12877-021-02623-x

**Published:** 2022-01-25

**Authors:** Anne Nobels, Adina Cismaru-Inescu, Laurent Nisen, Bastien Hahaut, Marie Beaulieu, Gilbert Lemmens, Stéphane Adam, Evelyn Schapansky, Christophe Vandeviver, Ines Keygnaert

**Affiliations:** 1 International Centre for Reproductive Health, Department of Public Health and Primary Care, Ghent University, Ghent, Belgium; 2 Department of Psychiatry, Ghent University Hospital, Ghent, Belgium; 3 CARE-ESPRIst, Études et évaluations, University of Liège, Liège, Belgium; 4 Psychology of Aging Unit, University of Liège, Liège, Belgium; 5 School of Social Work and Research Centre on Aging, University of Sherbrooke, Quebec, Canada; 6 Department of Head and Skin – Psychiatry and Medical Psychology, Ghent University, Ghent, Belgium; 7 Department of Criminology, Criminal Law and Social Law, Ghent University, Ghent, Belgium; 8 Research Foundation—Flanders (FWO), Brussels, Belgium

**Correction to: BMC Geriatr 21, 601 (2021)**

**https://doi.org/10.1186/s12877-021-02485-3**

After publication of this article [1], the authors reported that Christophe Vandeviver was not indicated as shared last author. Ines Keygnaert and Christophe Vandeviver are shared last authors.

The original article [1] has been updated.
