# Supplementary material for: Sexual violence in older adults: a Belgian prevalence study
Source: BMC Geriatr. 2021 Oct 26;21:601. doi: 10.1186/s12877-021-02485-3 (PMC8546387; doi:10.1186/s12877-021-02485-3)
Supplement: Supplementary file 1 — Additional file 1: Appendix 1. Detailed outcome measurements sexual victimisation. Appendix 2. Question on assailants of sexual violence. Appendix 3. Question on framing of sexual violence. Appendix 4. Question regarding coercion strategies. Appendix 5. Sociodemographic characteristics of the study population (n = 513) compared to the Belgian population of 70 years and older. [file 12877_2021_2485_MOESM1_ESM.docx]

# **Appendices**

**Appendix 1. Detailed outcome measurements sexual victimisation**

Hands-off sexual victimisation (no physical contact):

- *Sexual staring:* Someone stared at me in a sexual way or looked at my intimate body parts (e.g., breasts, vagina, penis, anus) when I didn’t want it to happen.
- *Sexual innuendo:* Someone made teasing comments of a sexual nature about my body or appearance even though I didn’t want it to happen.
- *Showing sexual images:* Someone showed me sexual or obscene materials such as pictures, videos, directly or over the internet (including email, social networks and chat platforms) even though I didn’t want to look at them. This does not include mass mailings or spam.
- *Sexual calls or texts:* Someone made unwelcome sexual or obscene phone calls or texts to me.
- *Voyeurism:* I caught someone watching me, taking photos or filming me when I didn’t want it to happen while I was undressing, nude or having sex.
- *Distribution of sexual images:* Someone distributed naked pictures or videos of me directly or over the internet (including email, social networks and chat platforms) when I didn’t want it to happen.
- *Exhibitionism:* Someone showed their intimate body parts (e.g., breasts, vagina, penis, anus) to me in a sexual way and/or masturbated in front of me when I didn’t want to see it.
- *Forcing to show intimate body parts:* Someone made me show my intimate body parts (e.g., breasts, vagina, penis, anus) online or face-to-face when I didn’t want to do it.

Hands-on sexual victimisation

Sexual abuse (physical contact but no penetration):

- *Kissing:* Someone kissed me against my will.
- *Touching in care:* Someone touched my intimate body parts (e.g., breasts, vagina, penis, anus) during care against my will.
- *Fondling/rubbing:* Someone fondled or rubbed up against my intimate body parts (e.g., breasts, vagina, penis, anus) against my will.
- *Forced undressing:* Someone removed (some of) my clothes against my will.

Rape and attempted rape (physical contact with attempted or completed penetration):

- *Oral penetration:* Someone had oral sex with me or made me give oral sex against my will.
- *Attempt of oral penetration:* Someone tried, but did not succeed, to have oral sex with me or tried to make me give oral sex against my will.
- *Vaginal or anal penetration:* Someone put their penis, finger(s) or object(s) into my vagina or anus against my will.
- *Attempt of vaginal or anal penetration:* Someone tried, but did not succeed to put their penis, finger(s) or object(s) into my vagina or anus against my will.
- *Forcing to penetrate:* Someone made me put my penis, finger(s) or object(s) into their (or someone’s) vagina or anus against my will.

**Appendix 2. Question on assailants**

Who was it that did this to you? (You may indicate multiple answers.)

1. My (ex)partner
2. Another family member
3. A friend
4. A date / someone I just met
5. Someone in position of authority (e.g., doctor, teacher, boss, police, religious worker, …)
6. A colleague / classmate
7. Someone else
8. Someone I didn’t know

**Appendix 3. Question on framing of sexual violence**

Do you consider this to be ...

1. A crime, a punishable offence
2. Wrong but not a crime
3. Just something that happened

**Appendix 4. Question regarding coercion strategies**

Which of the following circumstances describe best how this happened? (You may indicate multiple answers.)

1. Putting verbal pressure on you (e.g., by threatening to end the relationship, humiliating you, …)
2. Using or threatening to use physical force or harming you (e.g., holding you down, pinning your arms, …)
3. Exploiting the fact that you were unable to resist (e.g., because you had consumed too much alcohol or drugs or because you were incapacitated for some other reason)
4. Exploiting their position of authority or power over you
5. None of the above

**Appendix 5. Sociodemographic characteristics of the study population (n=513) compared to the Belgian population of 70 years and older.**

| Variable | | n (%) study population | % Belgian population ≥70y |
| --- | --- | --- | --- |
| Sex at birth | Female | 299 (58.3) | 57.5^a^ |
|  | Male | 214 (41.7) | 42.2^a^ |
| Age  (mean 79y) | 70-79y | 283 (55.2) | 58.3^a^ |
|  | 80-89y | 201 (39.2) | 34.5^a^ |
|  | 90-99y | 29 (5.7) | 7.2^a^ |
| Living situation | Community-dwelling | 462 (89.8) |  |
|  | Assisted living facility | 25 (4.9) |  |
|  | Nursing home | 27 (5.3) | 8.5^b^ |
| Country of origin | Belgium | 464 (90.4)^c^ | 94.0^c^ |
|  | Other | 49 (9.6)^c^ | 6.0^c^ |
| Education level | No formal education | 10 (1.9) | Higher education: 32.9^d^ |
|  | Primary education | 117 (22.8) |  |
|  | Secondary education | 116 (22.6) |  |
|  | Technical or vocational education | 109 (21.2) |  |
|  | Religious school | 1 (0.2) |  |
|  | Higher education | 160 (31.2) |  |
| Sexual orientation | Heterosexual | 475 (92.6) | 96.7^f^ |
|  | Non-heterosexual | 38 (7.4)^e^ | 3.3^f^ |
| Relationship status | Living together with partner | 225 (44.0) | Married: 52.8^a^ |
|  | Relationship, but living apart | 32 (6.3) |  |
|  | No relationship/ partner | 254 (49.7) | Unmarried/widowed/  divorced: 47.2^a^ |

^a^Numbers from Belgian Population Statistics 01/01/2019.
^b^% of Belgian population of 65 years and older receiving long term residential care in 2016.
^c^Numbers of Belgian Population Statistics describe nationality, while our questionnaire asked about country of birth.
^d^Numbers on education only available for Belgian population between 15 and 64 years old.
^e^This group contains participants who labelled themselves as: homosexual, bisexual, pansexual, asexual or other. In this last group, several participants labelled themselves as “normal”. Since it was not clear whether they had difficulties understanding the different terms defining sexual orientation or whether they indeed labelled their sexual orientation as “other”, we decided to classify these participants as non-heterosexual.
^f^Numbers based on Sexpert study (2011), a study on sexual health in Flanders, Belgium.
